# Mitochondria-Mediated Anticancer Effects of Non-Thermal Atmospheric Plasma

**DOI:** 10.1371/journal.pone.0156818

**Published:** 2016-06-06

**Authors:** Aigul Zhunussova, Elina A. Vitol, Boris Polyak, Sultan Tuleukhanov, Ari D. Brooks, Richard Sensenig, Gary Friedman, Zulfiya Orynbayeva

**Affiliations:** 1 Department of Surgery, Drexel University College of Medicine, Philadelphia, Pennsylvania, United States of America; 2 Department of Biophysics and Biomedicine, Al-Farabi Kazakh National University, Almaty, Kazakhstan; 3 Department of Electrical and Computer Engineering, Drexel University, Philadelphia, Pennsylvania, United States of America; 4 Department of Surgery, University of Pennsylvania, Philadelphia, Pennsylvania, United States of America; Roswell Park Cancer Institute, UNITED STATES

## Abstract

Non-thermal atmospheric pressure plasma has attracted great interest due to its multiple potential biomedical applications with cancer treatment being among the most urgent. To realize the clinical potential of non-thermal plasma, the exact cellular and molecular mechanisms of plasma effects must be understood. This work aimed at studying the prostate cancer specific mechanisms of non-thermal plasma effects on energy metabolism as a central regulator of cell homeostasis and proliferation. It was found that cancer cells with higher metabolic rate initially are more resistant to plasma treated phosphate-buffered saline (PBS) since the respiratory and calcium sensitive signaling systems were not responsive to plasma exposure. However, dramatic decline of cancer oxidative phosphorylation developed over time resulted in significant progression of cell lethality. The normal prostate cells with low metabolic activity immediately responded to plasma treated PBS by suppression of respiratory functions and sustained elevation of cytosolic calcium. However, over time the normal cells start recovering their mitochondria functions, proliferate and restore the cell population. We found that the non-thermal plasma induced increase in intracellular ROS is of primarily non-mitochondrial origin. The discriminate non-thermal plasma effects hold a promise for clinical cancer intervention.

## Introduction

Prostate cancer is the second leading cause of death from cancer in North American and European men [1]. It is a slow growing cancer, but as many other types of cancer, it is generally incurable once it reaches the metastatic stage [2]. Existing chemotherapies have severe side effects and do not provide a cure for advanced stages of the disease. There is an urgent need for novel medical approaches for treating tumor types which tend to easily develop resistance to chemo- and radiation therapies [3]. Non-thermal atmospheric pressure plasma has been recently identified as a potent technology for modulating the function of both prokaryotic and eukaryotic cells. Non-thermal is distinguished from thermal plasma based on the relative energetic levels of electrons and heavy species of the plasma [4]. Biomedical applications of non-thermal plasma include surface sterilization [5], wound healing and blood coagulation [6, 7], anti-bacterial treatment [8] and induction of cancer cells apoptosis [9–11], stimulation of proliferative activities of endothelial cells [12], anti-bacterial treatment [13, 14].

In biomedical applications, non-thermal plasmas are characterized by the type of discharge and method of applying the plasma products to cells and tissues. The types of discharges commonly used include dielectric barrier discharge (DBD), corona discharge, and gliding arc discharge [15]. Dielectric barrier discharge plasma is generated in the gap between two electrodes driven by *a*.*c*. voltage, typically in the kHz frequency range. One of the electrodes is insulated with a dielectric barrier with high breakdown strength which prevents spark formation in the discharge region, thus eliminating localized overheating. The two broad categories of plasma application modality are *direct* and *indirect* treatment. Direct plasma application is one in which the tissues or cells are in direct contact exposing the sample to both the chemical plasma products and the electric field used to generate the plasma, with cell lysis being the most drastic physical effect observed [16]. The indirect involves administration of plasma-treated liquids to cells and relies on the transfer of plasma-generated reactive species to the cells while eliminating the exposure of cells to electric field of plasma. The method of liquid-mediated indirect treatment appears to be more suitable for future clinical applications when a tumor may be not accessible for direct treatment in a patient.

To realize the full potential of non-thermal plasma treatment for cancer therapeutics, the exact mechanisms through which plasma causes cell death must be understood. It is also critical to study the side effects of non-thermal plasma on healthy cells. The primary goal of this work is to explore the effects of indirect non-thermal plasma generated by microsecond (pulse width) dielectric barrier discharge on mitochondria-mediated processes. The mitochondria orchestrate cell metabolism and signaling, and therefore, they are a promising target for cancer therapy [17]. Yet, it has been demonstrated that high doses of plasma induce apoptosis in other cancers due to massive generation of intracellular reactive oxygen species (ROS) [9, 18] and the mitochondria are one of the major intracellular sources of ROS [19]. These facts indicate that elucidating the mechanisms of non-thermal plasma effects on mitochondria is critical for learning how we can advance proof-of-concept demonstrations into a clinically-relevant method for cancer treatment.

A new antitumor drug or therapeutic treatment targeted only to cancer cells without affecting normal ones is the Holy Grail in cancer research. Achieving this kind of selectivity is very challenging which is why the side effects of chemo and radiotherapies remain a major problem. In this work, we compare the outcomes of non-thermal plasma treatment for metabolically different normal and cancerous prostate cells. It is reasonable to hypothesize that both normal and cancerous cells can be affected through the mitochondria-mediated mechanism to hopefully different extents.

## Materials and Methods

### Cell lines and growth conditions

Human prostate metastatic DU145 cells were purchased from ATCC at the available passage 60 and used up to passage 70 (Manassas, VA USA). Cells were maintained in RPMI 1640 growth medium supplemented with 10% FBS. Human primary prostate cells PrEC obtained at passage 2 from Lonza Inc. (Allendale, NJ USA) were maintained in manufacturer recommended PrEGM medium with all required supplements according to the manufacturer protocol except gentamicin and used by passage 5. Both cell lines were grown at 37°C and 5% CO_2_ atmosphere.

### Generation of atmospheric non-thermal plasma and cell treatment procedure

To generate non-thermal plasma we used the setup described elsewhere [9, 20]. Plasma was generated by applying alternating polarity pulsed (500 Hz –1.5 kHz) voltage of 20 kV magnitude (peak to peak), 1.65 μs pulse width and a rise time of 5 V/ns between the high voltage electrodes using a variable voltage and frequency power supply (Quinta, Russia). One mm thick quartz glass was used as an insulating dielectric barrier covering the 1-inch diameter copper electrode. A grounded mesh was placed between the high voltage copper electrode and the surface of the medium to block electrons and ions and allow only uncharged gas species to reach the medium.

The plasma dose for a DBD device is often expressed as energy per unit area (Joules/cm^2^), where energy is the output energy provided by the power supply (input to the electrode) and the area is the surface area of the electrode. Although this definition is useful in many applications it is not an actual measure of the plasma energy delivered to the medium. For our purposes it was more useful to characterize the plasma doses (D1-D7) through the shifts of pH values of 1 ml double deionized water in an eppendorf tube using 0.5 cm pH electrode tip upon application of plasma at certain frequency/power ratio and duration (**Table 1**). The narrowing 1 cm diameter opening of the eppendorf tube enables to minimize the contact of water surface with atmospheric CO_2_, which could buffer water pH.

**Table 1:** Determination of non-thermal plasma doses as a function of pH change.

Setup parameters	Non-thermal plasma doses						
	D1	D2	D3	D4	D5	D6	D7
**Hz:W:Sec**	2:3:5	2:3:10	2:3:15	2:3:20	2:3:30	3:3:20	3:3:30
**pH**	6.4±0.13	4.9±0.11	4.5±0.13	4.1±0.11	3.9±0.14	3.7±0.14	3.2±0.01

The plasma was applied to double deionized water at different frequency/power ratio and duration. The more acidic the water, the stronger the plasma dose.

The use of indirect plasma mediated through plasma-treated fluids including deionized water, phosphate-buffered saline (PBS), and N-acetyl-cysteine (NAC) has been evaluated for various purposes [21]. In this work the PBS (Ca^2+^/Mg^2+^ free) was chosen to produce plasma-treated solution as by composition it is similar to physiological solution used in clinical practices. The PBS treated with plasma dose D7 retained pH 6.8.

Cells monolayers were washed from growth medium and covered with plasma treated PBS for 1 or 10 minutes and the solution was diluted with fresh complete medium in 1:15 (plasma-PBS:RPMI medium) volume ratio.

### Cytotoxicity test

Cytotoxicity was assessed by the Alamar Blue assay [22]. Cells were seeded in the 96-well plate (10,000 cells/well). Control and experimental cells were incubated with 50 μl of 3% Alamar Blue solution prepared in a complete growth medium at 37°C for 2 hours. The fluorescence signal (peak emission 585 nm) was read on BioTek Synergy 4 microplate reader (Winooski, VT, USA).

### Colonogenic cell survival assay

To validate the antiproliferative effects of non-thermal plasma DU145 (30,000 cells) and PrEC (20,000 cells) were seeded in the 6-well plate and maintained overnight. Cells were exposed to 200 μL of plasma treated PBS (Dose 7) for 1 and 10 minutes and then neutralized by adding 3 mL of complete medium. Cells were cultured for 6 days. The colonies were fixed with 3.7% paraformaldehyde at room temperature for 5 minutes, rinsed with PBS, and then stained with 0.05% crystal violet for 30 minutes. After staining cells were washed with tap water and drained. The stained colonies were imaged on a Leica MZ16F stereomicroscope (Heerbrugg, Switzerland). The images were analyzed using ImageJ NIH software.

### Measurement of cell oxidative phosphorylation

The cell respiration was analyzed at 37°C in a two chamber respirometer OROBOROS Oxygraph-2K (Innsbruck, Austria) [23, 24]. Cells harvested by trypsinization and centrifugation were incubated with plasma treated PBS for 1 or 10 minutes, then diluted with fresh medium. Right after that or after 24 h of incubation of plasma treated cells in growth medium cells were sedimented at 1500 rpm for 5 minutes. Cell pellet was resuspended in a modified Krebs buffer containing 137 mM NaCl, 5 mM KCl, 20 mM MOPS, pH 7.4, 2 mM MgCl_2_, 1 mM KH_2_PO_4_, 2 mM CaCl_2_ and immediately measured.

### Evaluation of mitochondria membrane potential

Cells (0.2x10^6^) were loaded with 75 nM MitoRed (Ex/Em 622/648 nm) (PromoCell GmbH, Heidelberg, Germany) sensitive to mitochondria membrane potential. After sedimentation the cell pellets were mixed with pure Ca^2+^/Mg^2+^ free PBS or the plasma treated PBS (D7). Measurements were conducted on BD Accuri C6 flow cytometer (BD Biosciences, San Jose, CA). For the positive control cells were treated with 2 μM of FCCP, the dose which results in collapse of the membrane potential.

### Measurement of oxidative stress

Oxidative stress was assessed fluorimetrically using CM-H_2_DCFDA and MitoSox, hydrogen peroxide and mitochondria-generated superoxide sensitive dyes, correspondingly. Cells (0.2x10^6^) were loaded with 2 μM CM-H_2_DCFDA (Ex/Em 495/527 nm) or 5 μM MitoSox (Ex/Em 510/570 nm) (Life Technologies, Grand Island, NY). Changes in the level of H_2_O_2_ or superoxide were examined on BD Accuri C6 flow cytometer (BD Biosciences, San Jose, CA). For the positive control cells were challenged with 1μg/ml rotenone, 500 μM malonate, and 2.5μM antimycin, inhibitors of respiratory complexes I, II and III/IV, correspondingly.

### Assessment of apoptosis

Apoptosis induction in cells was verified using Annexin V-Propidium Iodide based apoptosis kit (Life Technologies, Grand Island, NY) and analyzed on BD Accuri C6 flow cytometer (BD Biosciences, San Jose, CA) using excitation and emission wavelengths at 488 and 525 nm, respectively.

### Confocal microscopy analysis

For microscopy experiments of cytosolic calcium modulations, cells were seeded on 35 mm MatTek glass bottom dishes (MatTek Corp., Ashland, Massachusetts) at a density of 200,000 cells/dish. The cells were rinsed from the growth medium with PBS (with Ca^2+^ and Mg^2+^) and labeled with 2 μM Fluo-4AM (Ex/Em 488/560 nm) for 15 minutes at room temperature in the dark. After labeling, the cells were washed twice and kept in the buffer for additional 15 minutes for stabilization. Cells were covered with 0.5 ml of PBS (Ca^2+^/Mg^2+^ free) and then either 1 ml pure PBS or plasma treated PBS were added to cells. The stability of cytosolic calcium level was monitored for 10 minutes before addition of 50 μM ATP. The cellular measurements were performed using Olympus FluoView^TM^ FV1000 confocal laser scanning inverted microscope, which allows simultaneous video imaging and micro-fluorimetry for monitoring intracellular fluorescence fluctuations.

### Western blotting

DU145 and PrEC cells (5x10^6^) were harvested and processed to obtain the whole cell lysates. After centrifugation at 2000 rpm for 5 minutes the cell pellets were resuspended in 1 ml of Ripa buffer containing protease inhibitors cocktail (Roche, Indianapolis, IN USA). Lysates were heated at 100°C, centrifuged at 14000 rpm for 10 minutes and subjected to 12% SDS-PAGE (30μg per sample) followed by transfer to nitrocellulose membrane and immunoblotting with corresponding antibodies, human anti-caspase 8, anti-caspase 9, and anti-caspase 3 (1:500) (Santa Cruz Biotechnology, USA), and anti-vinculin was used as a loading control (1:1000) (Cell Signaling, USA). HRP-linked secondary antibodies (anti-mouse 1:5000; anti-rabbit 1:3000) (Santa Cruz Biotechnology, USA) were visualized by chemiluminescence. Protein concentration was determined by Bradford Protein Assay (Bio-Rad Laboratories, USA).

### Statistical Analysis

Statistical analyses were performed using Prism program version 5.03 for Windows (GraphPad Software, San Diego, USA). Results are presented as mean ± SEM from at least three independent experiments. Statistically significant differences between data were estimated by unpaired, two-tailed Student's *t*-test. Differences were considered significant at *p* < 0.05.

## Results

### Plasma exerts distinct cytotoxicity on prostate cancer and normal cells

**Fig 1:**
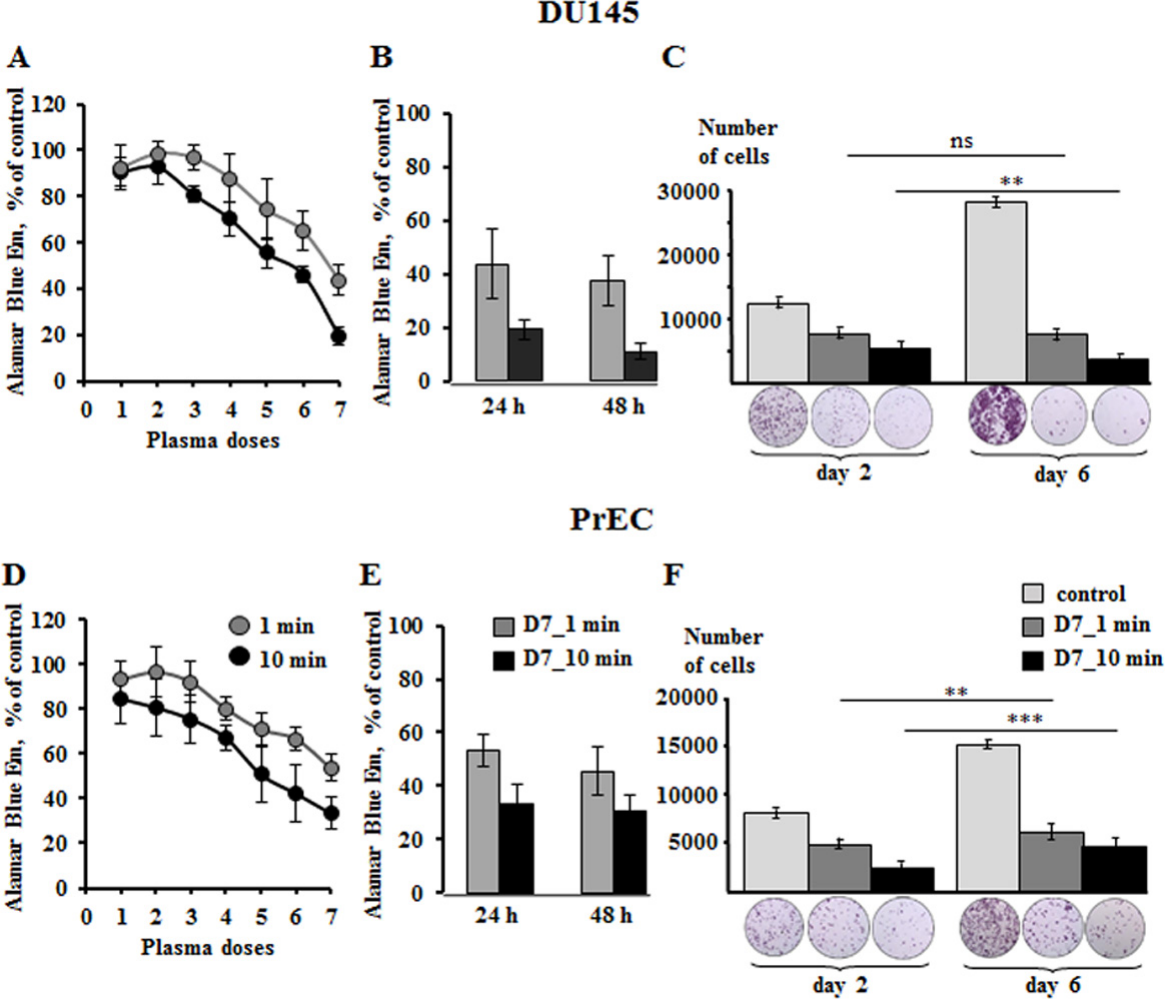
Cytotoxicity of non-thermal plasma on prostate DU145 cancer and PrEC normal cells. The cells were exposed to PBS treated with different plasma doses for 1 and 10 minutes, followed by dilution with growth medium and incubation for 24 and 48 hours. To simplify the understanding of non-thermal plasma doses, the plasma obtained at different physical parameters (**Table 1**) was named numerically as Dose 1, 2, etc. As seen from the graphs **A** and **D** the highest cytotoxic dose was Dose 7 (D7). (**B,E**) The quantitative graphs of the plasma induced cytotoxicity for both prostate cancer and normal cells. (**C,F**) The data and images obtained from the colony formation assay. The benign PrECs treated with plasma D7 for 1 and 10 minutes followed by PBS dilution with growth medium were cultivated for 6 days. They retain their proliferative activity while the cancer cells treated in the same way lose the ability to form colonies. Data presented as mean±SEM (n = 3).

To assess the effects of plasma on metabolism of metastatic prostate DU145 cells and their normal counterparts, human primary prostate epithelial cells PrEC, first we determined the plasma doses that are potentially cytotoxic. Alamar Blue assay was utilized to assess the dose-dependent plasma toxicity 24 hours after cell treatment with 7 different doses of plasma D1-D7, with dose 7 (D7) being the strongest one (**Table 1**). In this study we used the D7 only, varying its effect by applying it to cells for either 1 or 10 minutes and then diluting the plasma treated PBS with a complete cell growth medium. The cytotoxic effects of the same doses of plasma treated PBS were about 20% lower in normal than in cancer cells (**Fig 1A and 1D**). The plasma induced cell death increased over time reaching the values of 38% and 45% (in 48 hours) over 44% and 53% (in 24 hours) in DU145 cells and PrECs, correspondingly (**Fig 1B and 1E**).

Further, we validated the effects of plasma treated PBS on cell proliferative activity. Obviously, 10 minute exposure revealed a stronger suppression of cellular ability to form colonies than 1 minute exposure (**Fig 1C and 1F**). In contrast to cancer cells, normal PrECs showed tendency to restore their colony formation activity after treatment with both 1 and 10 minutes of plasma D7.

### Plasma induced apoptosis

**Fig 2:**
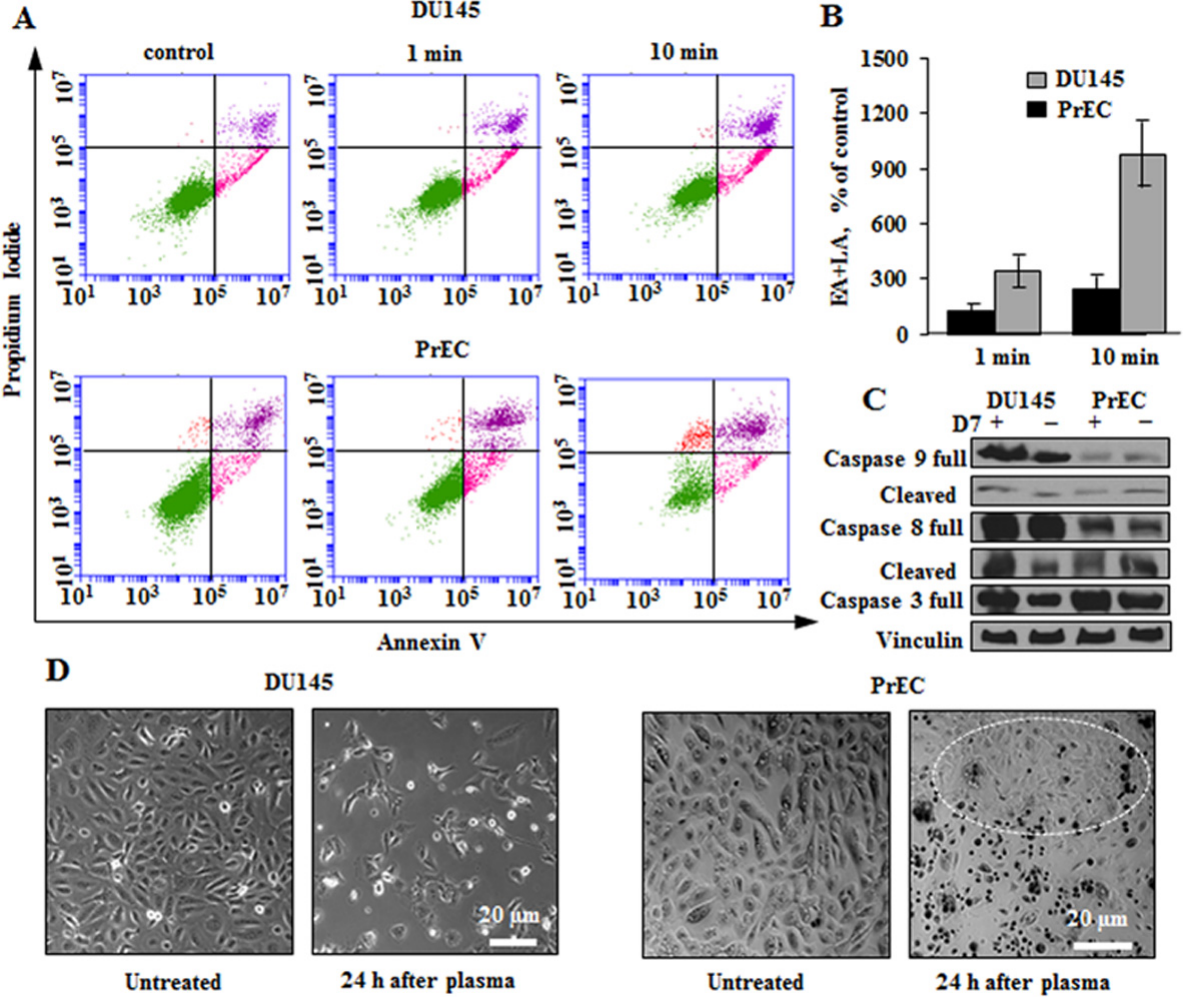
Non-thermal plasma induces apoptosis in DU145 cancer and PrEC normal prostate cells. Flow cytometry and microscopy results were obtained 24 hours post plasma treatment. (**A**) Induction of apoptosis in DU145 and PrECs. The cells were incubated with plasma D7 for 1 and 10 minutes than fresh medium was added to cells for their further maintaining. (**B**) The quantitative data of the per cent of early and late (EA+LA) apoptotic cells. **C.** Western blot analysis of apoptosis signature. Vinculin was used as a loading control. (**D**) Transmission images of normal PrEC and metastatic DU145 cells. The white circle indicates the area of PrECs that remained alive or proliferated after the plasma treatment. Data presented as mean±SEM (n = 3).

The **Fig 2**shows that plasma D7 induces apoptosis in both prostate normal and cancer cells. The 10 minute treatment with plasma D7 resulted in more pronounced induction of apoptosis reaching out 257% of control versus 143% for 1 minute treated PrECs (**Fig 2A and 2B**). However, DU145 metastatic cells demonstrated higher susceptibility to 10 minutes plasma treatment.

Late apoptosis was observed in cancer cells treated for 10 minutes by increase of 981% over control cells versus 335% for 1 minute treated cells (**Fig 2B**). The Western blot analysis revealed that DU145 cells undergo apoptosis by intrinsic and extrinsic pathways since both caspases 8 and 9 are profoundly present after plasma treatment (**Fig 2C**). The normal PrECs proceed to apoptosis mainly by non-mitochondrial cascade.

### Plasma induced alterations of mitochondria oxidative phosphorylation

**Fig 3:**
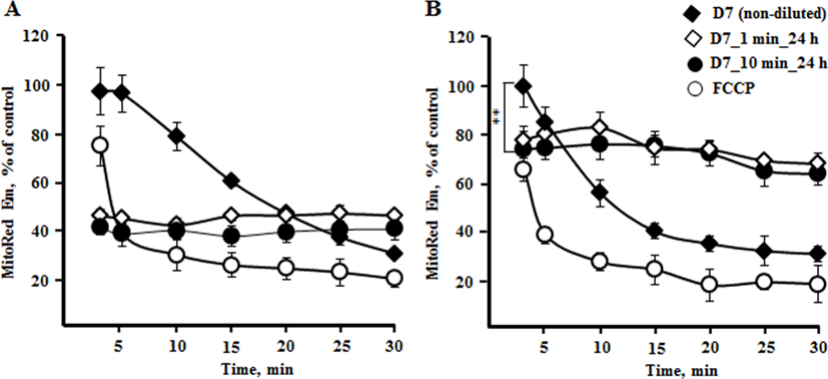
Effect of non-thermal plasma treatment on mitochondria membrane potential. The continuous exposure of cells to plasma treated PBS without it further dilution with medium results in a decay of mitochondria energization (**D7,** black rhombi) similarly to what was observed in the presence of protonophore (**FCCP,** empty circle). Decline in mitochondria energization in normal cells (**B,** black rhombi) was faster than in cancer cells (**A,** black rhombi), which are more resistant to plasma induced modulations. Samples **D7_1 min_24 h** (white rhombi) and **D7_10 min_24 h** (black circle) are the cells harvested after 24 h of incubation in the growth medium after being exposed to plasma D7 for 1 and 10 minutes. The normal cells restore their the membrane potential up to 70% within 24 h after treatment, while in the cancer cells the membrane potential remained at about 40% only (white rhombi and black circles). Data presented as mean±SEM (n = 3).

One of the key questions addressed in this work was related to the role of mitochondria in plasma induced alterations of cell homeostasis. The mitochondria are central for generation of ATP and reactive oxygen species for signaling purposes and in response to stress. First, we evaluated the effects of plasma D7 on mitochondria membrane potential (Δψ_m_) (**Fig 3**). Exposure of cells to plasma treated PBS without further dilution with medium results in drop of Δψ_m_ up to 30% of control in both cell types, approaching almost the collapse of membrane potential as it happens in the presence of 2 μM protonophore FCCP (carbonyl cyanide 4-(trifluoromethoxy) phenylhydrazone)

As seen in the **Fig 3**, during the first two minutes the plasma treated PBS did not cause a decrease in membrane potential of cancer cells, while it decreased the membrane potential of normal mitochondria up to 80% (black rhombi). The overall dynamics of decline of the level of Δψ_m_ in normal cells was sharper than in cancer cells indicating a stronger effect on normal cells.

**Fig 4:**
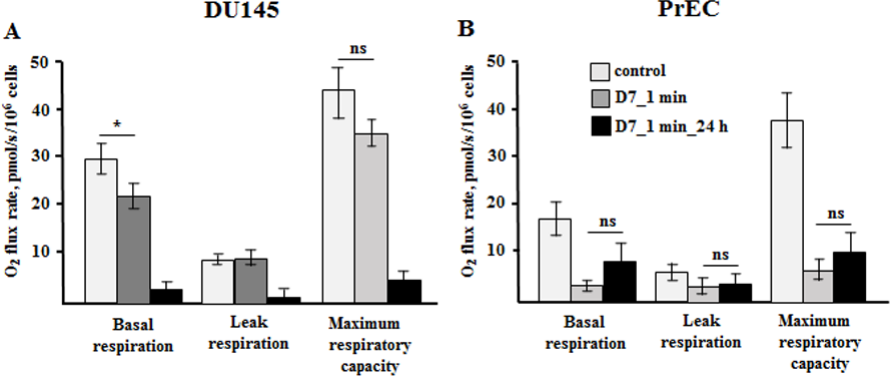
Respirometric analysis of non-thermal plasma effects on cell oxidative phosphorylation. Cells were measured in the respiratory buffer without respiratory substrates in order to evaluate the cell’s endogenous respiratory capacity. To evaluate the maximal respiratory activity the cells were inhibited with oligomycin to block ATP synthase and then titrated with increasing doses of protonophore. (**A,B**) Oxygen consumption rates in prostate cancer and normal cells immediately and after 24 hours post plasma treatment. Data presented as mean±SEM (n = 4–6).

After 24 hours post plasma treatment normal PrECs are capable of restoring their mitochondria membrane potential up to 70% of the control regardless of 1 or 10 min exposure time. The metastatic DU145 cells exposed to plasma treated PBS for 1 or 10 min retain the membrane potential after 24 h of incubation on the level of about 40% only. This decrease in mitochondria membrane potential reflects alterations in mitochondria oxidative phosphorylation (OxPhos) (**Fig 4**).

The plasma effects after 10 minutes exposure and following dilution with medium were more dramatic than after 1 minute exposure. The 1 minute exposure enabled us to follow the details of the mechanism of modulation of cellular processes, therefore in the following data only the effects of 1 minute plasma exposure are demonstrated.

It was interesting to find to what extent the effects of plasma vary in cells with elevated (DU145) and low (PrEC) metabolic activity. The basal respiration of cancer cells exposed for 1 minute to plasma treated PBS slightly decreased from 29.8±2.7 to 22.3±1.9 pmolO_2_/s/10^6^cells, although the proton leak and maximal electron transport respiration were not affected (**Fig 4A**). The PrECs immediately responded on plasma addition by decrease of bioenergetic parameters (**Fig 4B**). The mitochondria basal and maximal respiration rates decreased from 18.7±3.6 and 40.9±7.2 pmolO_2_/s/10^6^cells to 3.0±0.8 and 6.7±1.8 pmolO_2_/s/10^6^cells, correspondingly, while leak respiration did not change. However, in contrast to cancer cells, whose bioenergetic activities declined over 24 hours, the normal cells demonstrated slight tendency to restore their OxPhos activity consistent with the data in the **Figs 1F** and 2**D**.

### Oxidative stress induced by non-thermal plasma

**Fig 5:**
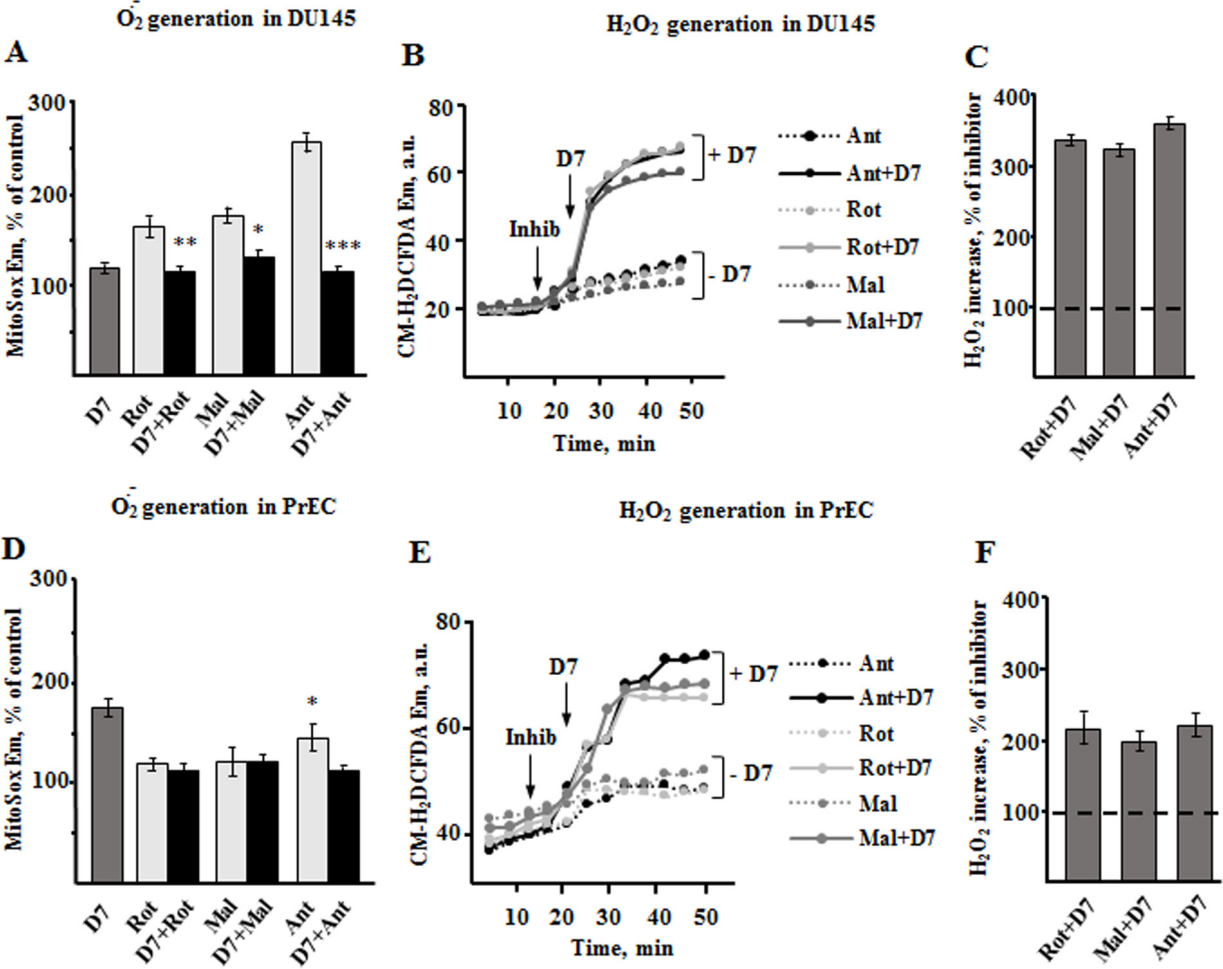
Non-thermal plasma induces oxidative stress in prostate cancer and normal cells. Flow cytometry measurements were performed in the presence of inhibitors of mitochondria respiratory complexes I (1μg/ml rotenone, Rot), II (500 μM malonate, Mal) and III/IV (2.5 μM antimycin, Ant). The inhibitors were used in combination with plasma treated PBS (D7) and dynamic changes of fluorescent probes’ emission was measured for 50 minutes. (**A,D)** Quantitative data of changes of MitoSox signal under modulation of cells with inhibitors and plasma treated PBS. The statistical significance is presented on top of the columns with respect to the plasma induced signal (D7). (**B,E)** Dynamic changes of CM-H_2_DCFDA emission upon stimulation of cells with inhibitors and plasma treated PBS. (**C,F)** Quantitative data of changes of CM-H_2_DCFDA signal. Data presented as mean±SEM (n = 3).

Previous studies demonstrated that the major effective components of non-thermal plasma are various reactive oxygen species produced during application of DBD [15, 25]. The mitochondria respiratory complexes are one of the intracellular sources of ROS due to probability of electron leakage. Therefore, we aimed at understanding the involvement of mitochondria, whose metabolism, as we demonstrated in experiments above, is unbalanced due to plasma exposure, in generation of plasma-mediated ROS, if any. Two fluorescent probes were employed, MitoSox, which is sensitive to mitochondria specific superoxide, and CM-H_2_DCFDA, which is sensitive to cytosolic H_2_O_2_. Using both dyes allows to single out mitochondria-specific plasma effects. The signals from both probes were evaluated in the presence of mitochondria respiration inhibitors, a useful tool for investigating the mechanisms of ROS generation [26, 27]. As seen from **Fig 5A**, addition of plasma treated PBS to DU145 cells induced elevation of superoxide release by 17% relative to background. Addition of the inhibitors of respiratory complexes I (rotenone), II (malonate), and III/IV (antimycin) over the plasma treated PBS increased superoxide release by 14%, 28%, and 16% only. In combination with plasma treated PBS, the inhibitors did not produce massive ROS release unlike in the case when inhibitors were used alone, and the superoxide release signal was 166%, 175%, and 262% of the control, correspondingly (**Fig 5A**). To test the possible mock-sensitivity of MitoSox to other reactive oxygen species that could manifest the observed signal and also the effects related to plasma induced mitochondria deenergization, we measured the changes of MitoSox emission in response to 2μM FCCP and 100μM H_2_O_2_. We found that these two agents do not alter the probe signal and do not produce any changes in a background emission.

In contrast to cancer cells, the normal prostate cells were shown to be more sensitive to the non-thermal plasma effects as the level of mitochondria-mediated superoxide increased by 76% over control (**Fig 5D**). This value is much higher than that caused by inhibitors rotenone, malonate, and antimycin alone (by 17%, 19%, and 46% over control).

Next, we evaluated the contribution of mitochondria to overall cell oxidative stress by measuring the CM-H_2_DCFDA signal. As seen from **Fig 5B**, the mitochondria inhibitors given in concentrations that induce strong superoxide release, caused only slight increase of CM-H_2_DCFDA signal in DU145 cells. Addition of plasma treated PBS dramatically increased the signals produced by inhibitors used, rotenone, malonate, and antimycin, up to 333%, 317%, and 350%, correspondingly (**Fig 5B and 5C**), indicating the non-mitochondria origin of bulk plasma-mediated ROS.

Addition of plasma treated PBS to benign PrECs in the presence of respiratory inhibitors induced H_2_O_2_ production by 216%, 194%, and 222% over H_2_O_2_ signal induced by inhibitors alone. (**Fig 5E and 5F**). Thus, in PrEC benign cells the main source of plasma induced ROS are not mitochondria.

### Plasma effects on cytosolic calcium oscillations

**Fig 6:**
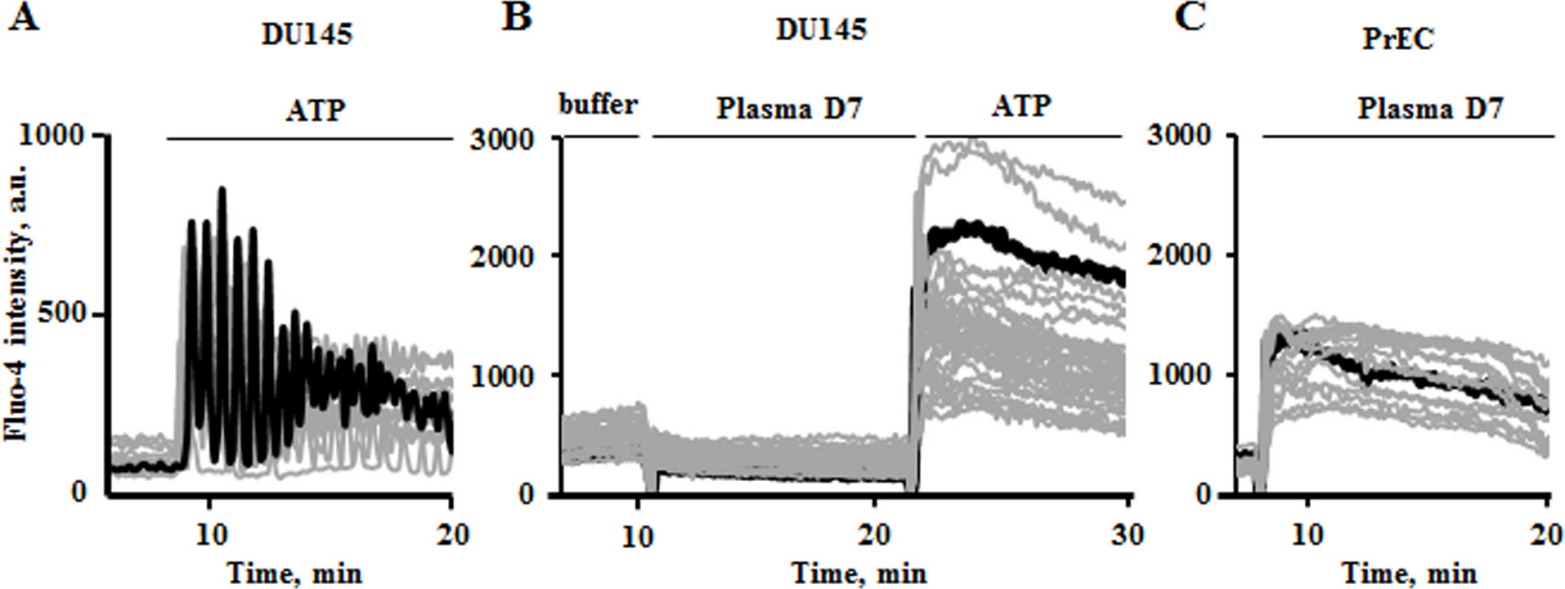
Plasma induced modulations of cytosolic calcium in PrEC and DU145 cells. Representative confocal microscopy spectral records of DU145 cells. After 10 minutes of incubation with or without plasma treated PBS cells were challenged with 50μM ATP. (A) IP_3_-mediated intracellular calcium oscillations induced by ATP were produced in control cells. (B) In plasma treated DU145 cells this amount of ATP provoked sustained cytosolic calcium response, while non-thermal plasma treated PBS itself did not cause calcium modulations. In PrECs the high calcium signal was observed right after addition of non-thermal plasma treated PBS (D7) (C). The representative original records demonstrate the data collected from about 50 cells evaluated in each of 5 experiments.

One of the common cellular responses to different stress factors is the fluctuation of cytosolic calcium. Yet, mitochondria are known to be the key regulators of calcium homeostasis being capable of sequestering large amounts of Ca^2+^ to protect cells from calcium overdose. We found that DU145 cells do not respond to indirect plasma treatment by Ca^2+^ elevation. During 10 minutes of exposure to plasma treated PBS DU145 cells did not produce any measurable effects on cytosolic calcium signal (**Fig 6B**), while in PrECs, the cytosolic calcium increased immediately after addition of plasma treated PBS (**Fig 6C**). Importantly, the plasma treated PBS was Ca^2+^ and Mg^2+^ free, therefore the calcium signal observed in cells is originated from the internal reservoirs. To test responsiveness of calcium signaling system of DU145 cells, we challenged them with 50μM ATP to stimulate the calcium dependent IP_3_-signaling (**Fig 6B**). Addition of ATP to plasma untreated cells produces frequent Ca^2+^ oscillations attenuating over time (**Fig 6A**). When ATP was added to DU145 cells that were incubated with plasma treated PBS, the sustained increase of cytosolic Ca^2+^ was detected (**Fig 6B**).

## Discussion

The major aim of this work was to study the prostate cancer specific plasma effects with a special focus on the role of mitochondria metabolism and ROS generating machinery in the mechanisms of plasma induced cell damage. The prostate tumor cells, unlike the most malignant tissues, are characterized by a low rate of glycolysis and glucose uptake [28] and rely on OxPhos. From our previous work we know that DU145 cells have greater mitochondria content and higher respiratory activities than their normal counterparts; yet, cancer cells are resistant to apoptosis due to higher mitochondria membrane potential and larger calcium retention capacity [29, 30]. This metabolic difference underlies the distinct responses of prostate cancer and normal cells to plasma induced stress. Thus, although benign cells are sensitive to plasma damaging effects (**Fig 1**), they clearly demonstrated signs of the ability to recover and proliferate over time (**Fig 1F**). Incubation of PrECs for 24 hours with plasma treated PBS induced apoptotic death, but large groups of cells were observed to have normal morphological features. These cells assured replacement of the cell population over time (**Fig 2E**). Plasma treatment induces apoptosis both in DU145 and PrEC cells, although in cancer cells, both mitochondria-mediated and death-receptor supported death pathways are triggered (**Fig 2**).

Since the active components of non-thermal plasma treated liquids are shown to be highly harmful reactive oxygen and nitrogen species [9, 18], we questioned the contribution of mitochondria, one of the key cell metabolic regulatory centers and physiological sources of ROS, in the development of oxidative stress caused by non-thermal plasma.

We demonstrated that exposure of cancer and normal cells to non-thermal plasma treated PBS reduces mitochondria membrane potential (Δψ_m_). However, 24 hours after treatment the normal cells maintain Δψ_m_ at the level much higher than the cancer cells, 70% versus 40% (**Fig 3**). The elevation of membrane potential to that level enabled the survived PrECs to slightly recover respiration within 24h hours, although the values were shown to be non-significant. Due to the inherently distinct metabolism of cancer and normal prostate cells, non-thermal plasma treated PBS affected their respirometric functions antithetically. Addition of plasma treated PBS did not cause a drop of cancer respiration immediately. However, after 24 hours OxPhos in DU145 cells declined dramatically correspondingly to the Δψ_m_ level (**Figs 3A** and **4A**). In contrast, normal cells in the presence of plasma treated PBS revealed suppressed basal respiration (**Fig 4B**). It could be a result of either one or both the decline of mitochondria membrane potential due to plasma-mediated membrane perturbations (**Fig 3B**) and the massive increase of cytosolic calcium (**Fig 6**), which activates the calcium sequestrating activity of mitochondria also at cost of membrane potential. However, after 24 hours the normal cells show signs of recovering of the respiration.

The metabolic state of mitochondria determines the rates of ROS generation by these organelles. The contribution of mitochondria originated ROS to non-thermal plasma induced ROS is one of the questions we addressed in this work. In the case of indirect plasma treatment employed in this work, the major plasma effector on cells is reactive oxygen and nitrogen species that enrich the cytosolic pool of those species upon cell exposure. At the same time, the possible direct targeting of mitochondria constituents by plasma could also result in deleterious elevation of cytosolic ROS. The slowing of electron flow by decreased membrane potential, other membranal interruptions and alteration of variety of mitochondrial matrix dehydrogenases could facilitate electron leakage and elevation of superoxide production [31, 32]. It must be noted that the prostate cancer cells naturally have higher ROS background which is actually due to non-mitochondrial sources [33], so the ROS threshold in cancer cells upon plasma treatment is expected to be lower. As seen from the **Fig 5**, the non-thermal plasma increases the level of superoxide production in cancer and normal cells.

We assessed the role of specific respiratory chain sites of ROS generation, namely complexes I, II, and III, in plasma induced effects. The complexes I and III are classical sites of electron leakage [27]. However, the complex II impact on ROS production by other complexes has started to attract attention, too [26, 34–36]. The useful tools for studying the mechanisms of ROS formation are the inhibitors of enzymatic complexes. Rotenone causes reduction of the Coenzyme Q site of complex I preventing forward electron flow [37]. Under these condition the production of ROS at complex I occurs also due to a backward electron flow from the complex II. Malonate inhibits the succinate binding site of complex II [36]. The rationale for testing malonate was also the fact that non-thermal plasma treated PBS decreases the mitochondria membrane potential (**Fig 3**), and the complex II does not depend on mitochondria energization and can produce ROS at low membrane potential. Antimycin reduces the Coenzyme Q site of complex III [38]. In DU145 cells these inhibitors produce much higher superoxide formation than in the presence of plasma treated PBS (**Fig 5A**), and addition of inhibitors over the plasma treated PBS rather eliminated the plasma effect. Exposure of cancer cells to the plasma treated PBS increases the background superoxide only by 16% over control. In normal cells exposed to plasma treated PBS we observed higher superoxide production than in those exposed to inhibitors alone and in combination with plasma treated PBS (**Fig 5D**). This fact also calls for a question whether the non-thermal plasma produced ROS has sole origin in mitochondria. We validated the plasma effects using cytosolic H_2_O_2_-sensitive probe also. Non-thermal plasma treated PBS generates different active species including hydrogen peroxide. The uncharged molecules of H_2_O_2_ easily penetrate the cell membrane enriching the endogenous pool of hydrogen peroxide and along with other plasma-derived species further initiate the production of various intracellular ROS, including hydrogen peroxide. Similarly to what was demonstrated in earlier works [9, 39, 40], we detected a strong oxidative stress induced by plasma in studied cells (**Fig 5B–5F**). But the level of H_2_O_2_ stimulated by inhibitors alone was very low in comparison to what was produced by plasma treated PBS over the inhibitors. Addition of plasma treated PBS to the cells dramatically increased the level of bulk cytosolic H_2_O_2_ in both cell types indicating deleterious enhancement of ROS by the mechanisms that do not discriminate cancer and benign metabolic differences. Thus, the moderate reduction of membrane potential induced by plasma results in ROS production by mitochondria that to some extent enriches the pool of cell oxidative species, but in general the oxidative stress at given conditions primarily has a non-mitochondrial origin. The mitochondria vulnerability to ROS generated by non-thermal plasma [41] could be the later mechanism of mitochondria injury especially of prostate malignant mitochondria that are known to have an elevated workload.

Since the efficacy of energetic and many other cellular processes is tightly regulated by cytosolic Ca^2+^, we tested the effects of plasma exposure on changes of cytosolic calcium which is also linked to mitochondria OxPhos (**Fig 6**). Induced prolonged cytosolic calcium elevation observed in normal cells results in mitochondria malfunctioning and other intracellular injuries. Interestingly, cytosolic calcium level of prostate cancer cells does not change upon addition of plasma treated PBS, however, cell calcium signaling systems, including calcium sensitive and voltage gated channels and other cellular protein and membrane constituents, seem to get sensitized to extracellular stimuli by plasma exposure. Stimulation of plasma treated cells with ATP that binds to purinergic receptors resulted in sustained cytosolic calcium elevation. The mechanisms of such increased sustainability of cancer cells to plasma treatment need to be investigated yet.

In this work we demonstrated that the high energy metabolism and higher oxidative background of the prostate cancer cells causes them to be more vulnerable to plasma induced injury. Although normal cells are also very sensitive to the damaging effects of plasma treated PBS, plasma-mediated alterations were not lethal to normal cells which retained their ability to restore their metabolism in contrast to cancer cells for which lethality rate increased over time. The major cause of plasma induced damage is oxidative stress in which the mitochondria have been shown to play a secondary role. The data on different effects of non-thermal plasma on metabolically distinct malignant and normal cells hold great promise for future clinical application of non-thermal atmospheric dielectric discharge plasma for cancer intervention.

## Abbreviations

OxPhos: oxidative phosphorylation
FCCP: carbonyl cyanide 4-(trifluoromethoxy) phenylhydrazone
ROS: reactive oxygen species
DBD: dielectric barrier discharge
